# *Methoprene-tolerant* (*Met*) and *Krüpple-homologue* 1 (*Kr-h1*) are required for ovariole development and egg maturation in the brown plant hopper

**DOI:** 10.1038/srep18064

**Published:** 2015-12-14

**Authors:** Xinda Lin, Yun Yao, Bo Wang

**Affiliations:** 1 College of Life Sciences, China Jiliang University, Hangzhou, China, 310018

## Abstract

The brown plant hopper is one of the most destructive known pests of rice. We studied the roles of the JH receptor Met and the downstream transcription factor Kr-h1 in ovariole development and egg maturation. The predicted Met protein in *N. lugens* (NlMet) contained 517 amino acids. qRT-PCR showed that *NlMet* was expressed in all tissues and that the highest expression occurred in the embryonic stage. In *NlMet-* or *NlKr-h1*-silenced female adults, ovarian development varied significantly, whereas the numbers of ovarioles were less variable in those injected with dsRNA targeting *NlMet, NlKrh-1* or both *NlMet* and *NlKr-h1*. In females injected with dsNlKr-h1 or with dsNlMet in combination with dsNlKr-h1 dsRNA, the preoviposition period was prolonged, whereas the females injected with *NlMet* dsRNA showed no significant changes. Moreover, we found no differences in the length of the preoviposition period between macropterous and brachypterous females. The disruption of *Nlmet* or *NlKr-h1* or the dual knockdown of *NlMet* and *NlKr-h1* significantly reduced the number of eggs laid. Moreover, significant differences were also found between the macropterous and the brachypterous brown plant hoppers. These results indicated that Met and Kr-h1 are required for ovariole development and egg maturation in the brown plant hopper.

The brown plant hopper *Nilaparvata lugens* Stål is dimorphic and is one of the most destructive known pests of rice. The yield losses caused by the brown plant hopper are severe in several countries, including China, India, Indonesia, Japan, South Korea, the Philippines, and Sri Lanka, and infestations are also observed in many other countries^1^,^2^. The brown plant hopper is a serious threat to rice production in Asia. The brown plant hopper feeds directly on rice tillers and causes the rice to turn yellow, which ultimately leads to “hopper burn” when the pest population is large. This pest also transmits viruses, such as the virus causing grassy stunt disease and the rice stripe virus (RSV), which increase yield loss^3^,^4^.

The brown plant hopper is dimorphic, and typically, the wings develop such that there are long-winged macropterous forms and short-winged brachypterous forms. In the laboratory, the macropterous and brachypterous forms develop in similar proportions. However, in the field, an increase in the formation of macropterous forms typically indicates migration and leads to colonization of new fields. After colonization, the proportion of brachypterous forms increases. The brachypterous females are the primary reproductive form and typically lay more eggs than the macropterous females.

The signalling mediated by juvenile hormone (JH) is one of the key signalling pathways that controls insect development and normal adult physiological function^5^. The basic-helix-loop-helix/Per-Arnt-Sim (bHLH-PAS) domain protein *methoprene- tolerant* (*Met*) is the receptor for the JH ligand, and this domain contains the specific binding domain of the JH ligand-binding site (charles 2011; Miura 2005). *Met* forms a complex with another bHLH-PAS domain protein, *Tai/SRC/FISC* (*Taiman* (*Tai*), as well as with *steroid receptor coactivator* (*SRC*) and *Ftz-F1-interacting steroid receptor coactivator* (*FISC*))^6^,^7^,^8^,^9^. In mosquitoes, *Met* forms a complex with Cycle to regulate the circadian expression of JH-induced genes^10^. *Krüppel-homologue 1* (*Kr-h1*) is a zinc finger protein that acts downstream of the JH receptor and is expressed in response to JH signalling. *Kr-h1* is antimetamorphic and is required for the development of wings and external genitalia^11^. *Kr-h1* also participates in neural development and maturation^12^,^13^, in addition to maintaining normal physiological function in adults.

The signalling mediated by JH is a key pathway in the regulation of insect metamorphosis^5^, cellular immunity^14^, and trehalose homeostasis^15^. In *Drosophila melanogaster*, JH regulates female mating and pheromone production^16^ and male reproduction^17^. JH signalling is involved in vitellogenesis and oogenesis in female reproduction^18^. In *Tribolium*, the JH receptor *TcMet* is required for egg production and vitellogenin expression^19^. In *Aedes aegypti*, *Met* is required for ovarian development^20^ and egg production^7^. More recently, in *Pyrrhocoris apterus*, the silencing of *Met* or *Tai* blocked the development of the ovaries, whereas the silencing of *Kr-h1* did not^21^. However, in *Locusta migratoria*, the silencing of *Kr-h1* blocked oocyte maturation and ovarian development^22^. In our previous study with *N. lugens*, treatment with JH III or the analogues methoprene or pyriproxifen induced the expression of the downstream transcription factor Kr-h1^11^. Similar induction was reported in *Pyrrhocoris apterus*^23^, *Bombyx mori*^24^,^25^, *Drosophila melanogaster*^26^, *Tribolium castaneum*^27^ and *Aedes aegypti*^10^,^28^.

Understanding the development of the ovary is essential for making predictions about migratory insect pests. The development and maturation of eggs is also the basis of pest outbreaks. The development of the ovary of *N. lugens* has five stages: I. Undeveloped ovarioles. II. Developed ovarioles with immature eggs. III. A few ovarioles containing full-grown eggs. IV. More of the ovarioles containing full-grown eggs, and vacuity appears in some ovarioles. V. Ovarioles become wethered^29^,^30^. The stage of ovarian development of *N. lugens* is affected by various environmental factors and the developmental stage of the rice^29^,^31^. In *N. lugens*, JH is closely associated with migration, and the stage of ovarian development is significantly affected by JH and the antijuvenile hormone prococeneII^29^,^32^. However, the molecular mechanism underlying the effects remains unclear. In this study, we examined the roles of the JH receptor *Met* and the downstream transcription factor *Kr-h1* and their interaction during ovariole development and egg maturation in the hemimetabolous brown plant hopper.

## Results

### Phylogenetic analysis of the Nilaparvata lugens Methoprene-tolerant (Met) gene

**Figure 1:**
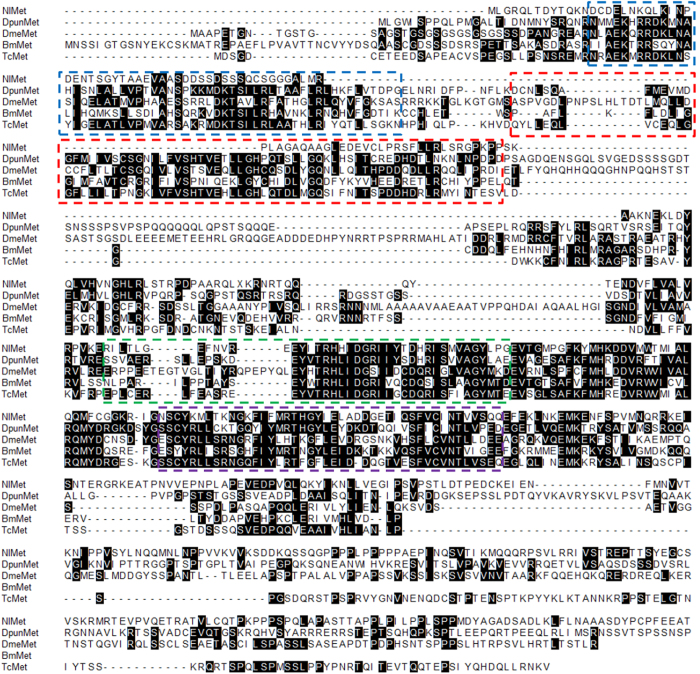
Alignment of the amino acid sequence of *N. lugens* Met with those of its homologues in *Bombyx mori*, *Diploptera punctata*, *Drosophila melanogaster*, and *Tribolium castaneum*. Domains—bHLH (Blue), PAS-A (Red), PAS-B (Green) and PAC (Purple)—are boxed. Bm, *Bombyx mori* (ACJ04052.1); Dpun, *Diploptera punctata* (AIM47235.1); Nl, *Nilaparvata lugens* (AIE12451.1); Tc, *Tribolium castaneum* (NP_001092812.1).

We performed a BLAST search in the transcriptome database and then cloned the *N. lugens methoprene-tolerant* (*NlMet*) gene. The *N. lugens NlMet* contains 2040 bp in the coding region and encodes 680 amino acids. The nucleotide sequence and the predicted amino acid sequence were deposited in NCBI Genbank (accession number: KP797880). We aligned the predicted protein sequence of *N. lugens Met* (*NlMet*) with homologues from *Diploptera punctata, Drosophila melanogaster, Bombyx mori* and *Tribolium castaneum*. The *NlMet* protein had typical bHLH, PAS-A, PAS-B and PAC (PAS C terminal motif) domains throughout the protein (Fig. 1).

Based on the phylogenetic analysis, the NlMet protein was highly conserved and clustered with the DpunMet protein, and these two proteins clustered with RpMet and PaMet (Figure S1).

### Spatial and temporal expression of *NlMet* during *N. lugens* development

**Figure 2:**
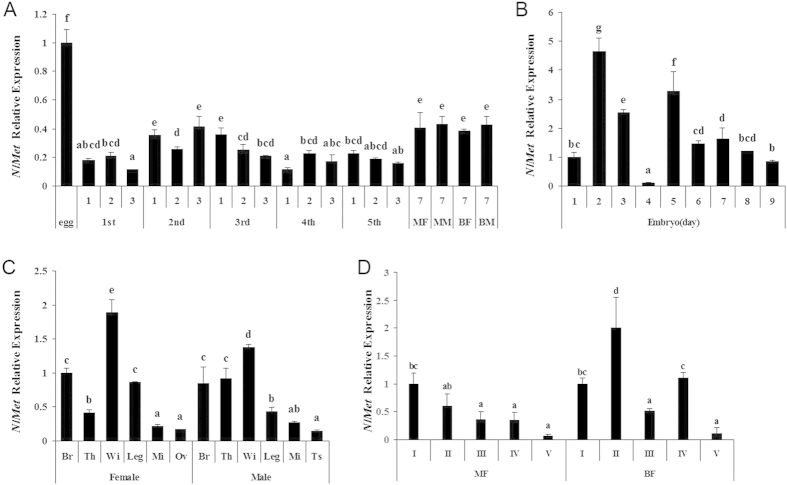
Spatial and temporal expression of NlMet. Expression during the embryonic development of the brown plant hopper (1^st^–9^th^ days, **B**), at each of the nymph stages (1^st^ to 5^th^, **A**) and in adults of the two wing forms (**A**). Also shown is the expression of *NlMet* in various tissues of the female and male (**C**). The relative expression of *NlMet* in the 1^st^ to 5^th^ stage ovaries of the brachypterous and macropterous females is shown in (**D**) The letters a–e indicate significant differences at a 0.05 level according to Duncan’s multiple range tests Macropterous female (MF), Macropterous male (MM), Brachypterous female (BF), Brachypterous male (BM), Adults (**A**). Tissue-specific expression of NlMet in the brain (Br), thorax (Th), wing (Wi), leg (Leg), midgut (Mi), fat body (Fb), ovary (Ov) and testis (Ts) of adult *N. lugens* (**C**).

The spatial and temporal expression of a gene effectively indicates the function of the gene. We measured the expression of *NlMet* at different developmental stages. The *NlMet* gene was expressed at all stages. The highest expression was in the embryonic stage (egg), whereas the expression was variable in the nymphal stage and was relatively constant in adults (Fig. 2). In the embryonic stage, the expression of *NlMet* was relatively high on the 2^nd^, 5^th^ and 3^rd^ days, but the expression was relatively low on the 4^th^ day (Fig. 2). The levels of expression were similar on the other days (Fig. 2). The expression of *NlMet* was also determined in various tissues, including the brain (Br), thorax (Th), wing (Wi), leg, midgut (Mi), ovary (Ov) and testis (Ts). In the midgut, ovary and testis, the expression of *NlMet* was relatively low, whereas the expression was relatively high in the wing and brain (Fig. 2). We also measured the expression of *NlMet* during the development of the ovaries of brachypterous and macropterous *N. lugens*. *NlMet* was expressed from the 1^st^ to the 4^th^ stage of ovarian development, but the expression decreased significantly in the 5^th^ stage of development in both the brachypterous and macropterous forms (Fig. 2D).

### Silencing of *NlMet* and *NlKr-h1* through RNAi in the nymphal stage of *N. lugens* disrupted the development of the ovariole

**Figure 3:**
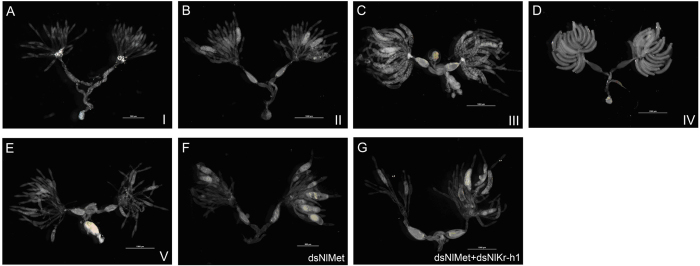
The stages I to V of the ovaries of *N. lugens* females (A–E) and the ovaries that did not fit the grading criteria (F,G) after silencing of *NlMet* alone or the dual knockdown of *NlMet* and *NlKr-h1*.

To test whether the development of ovarioles and the maturation of eggs required components of JH signalling, 5^th^ instar nymphs were injected with dsRNA or the control dsGFP. The ovaries were dissected, and the phenotypes were observed. For 7.69% of the ds*NlMet* adults and 6.9% of the adults that underwent dual knockdown of *NlMet* and *NlKr-h1*, the stage of ovary development could not be determined (Fig. 3F,G), because these ovaries either fit criteria for more than one stage of development or one of the two ovaries had fewer mature oocytes.

**Figure 4:**
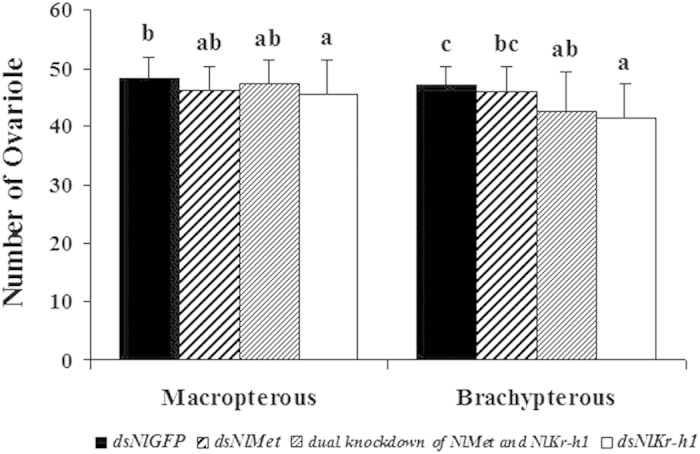
Effect of silencing NlMet and NlKr-h1 on the ovariole number of N. lugens females. The letters a–e indicate significant differences according to Duncan’s multiple range tests at a 0.05 level. The numbers of female adults used for the experiment: dsGFP (n = 43 brachypterous, 42 macropterous), dsNlMet (n = 42 brachypterous, 44 macropterous), dual knockdown of *NlMet* and *NlKr-h1* (n = 46 brachypterous, 37 macropterous), and dsNlKr-h1 (n = 38 brachypterous, 41 macropterous).

We then determined whether the “normal” remaining ovaries had equivalent numbers of ovarioles and similar developmental rates. The number of ovarioles was not significantly reduced in the *NlMet*-silenced macropterous or brachypterous females compared with that of the control female adults injected with dsGFP. However, in the *NlKr-h1*-silenced females and the *NlMet* and *NlKr-h1* double-silenced females, the numbers of ovarioles were reduced significantly compared with the control dsGFP brachypterous females (Fig. 4). In the macropterous females, the difference in the numbers of ovarioles between the females with dual knockdown of *NlMet* and *NlKr-h1* and the control was not significant, whereas the difference was significant when the dual knockdown females were compared with the *NlKr-h1*-silenced females (P < 0.05; Fig. 4).

**Figure 5:**
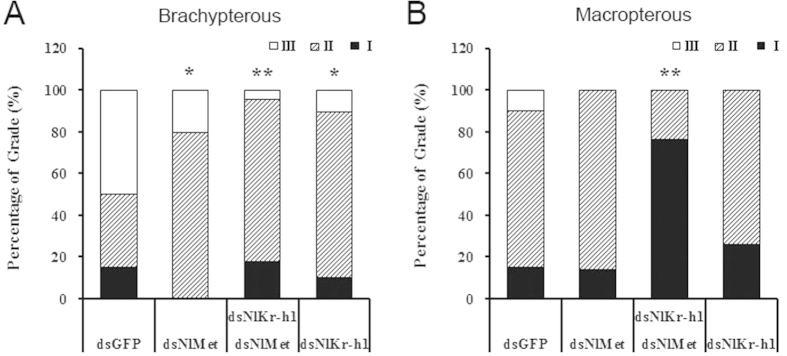
Effect of silencing NlMet and NlKr-h1 on ovariole development of N. lugens females. (**A**) Brachypterous; (**B**) Macropterous. Percentages of stages I, II, and III of ovariole development 2 days post-adult eclosion (PAE) are shown. For the Chi-square test, *P < 0.05 and **P < 0.01 compared with injection with dsGFP as the control. See Fig. 4 for the numbers of females used in the experiment.

To study the effect of gene silencing on the development of the ovariole, we recorded the stage of ovarian development in the adult females with dual knockdown of *NlMet* and *NlKr-h1*. Based on the grading criteria reported previously^29^, we measured the level of ovarian development 2 days after adult emergence. All the dsRNA-injected females had delayed ovarian development, which resulted in the delayed maturation of eggs. In the *NlMet*- or *NlKr-h1*-silenced brachypterous females, the development of the ovaries was slowed significantly compared with that of the control (P < 0.05; Fig. 5); it was slowed further in the females with the dual knockdown of *NlMet* and *NlKr-h1* (P < 0.01; Fig. 5). In contrast to the brachypterous form, the rate of ovarian development in macropterous females was not significantly different between the *NlMet* or *NlKr-h1-*silenced females and the dsGFP control. Notably, the rate of ovarian development was significantly slowed upon dual knockdown of *NlMet* and *NlKr-h1* (P < 0.01; Fig. 5).

### The roles of *NlMet* and *NlKr-h1* in maturation and the number of eggs

**Figure 6:**
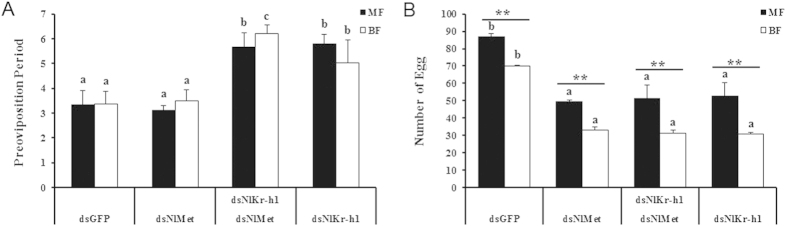
Effect of silencing NlMet and NlKr-h1 on the preoviposition period and the number of eggs of N. lugens females. (**A**) Preovipositon period; (**B**) Number of eggs laid. Comparison between macropterous and brachypterous adults for each dsRNA treatment, based on t-tests, P < 0.01. Comparison among different treatments, based on Duncan’s multiple range tests, P < 0.05. The letters a–e indicate significant differences according to Duncan’s multiple range tests at a 0.05 level. The numbers of females used for the experiment: dsGFP (n = 15 brachypterous and 17 macropterous), dsNlMet (n = 33 brachypterous and 14 macropterous), dual knockdown of NlMet and NlKr-h1 (n = 27 brachypterous and 6 macropterous), and dsNlKr-h1 (n = 22 brachypterous and 17 macropterous).

The preoviposition period occurs between emergence and the time the first eggs are deposited. To further investigate the roles of *Nlmet* and *NlKr-h1* in the development of ovaries and the maturation of eggs, we measured the preoviposition period of the *Nlmet-* and *NlKr-h1*-silenced females and the females with dual knockdown *NlMet* and *NlKr-h1*. The preoviposition period was prolonged in both the macropterous and the brachypterous females that were injected with *dsNlKr-h1* or *dsNlmet* and *dsNlKr-h1* dsRNA, whereas for the females injected with *NlMet* dsRNA, the preoviposition period was not different from that of the control (Fig. 6A). Moreover, the preoviposition period was not different between the macropterous and the brachypterous females upon any treatment or in the controls.

The number of eggs laid is an important indicator of an outbreak of an insect pest. We found that disruption of *Nlmet* and *NlKr-h1* and dual knockdown of *NlMet* and *NlKr-h1* significantly reduced the number of eggs laid (P < 0.05; Fig. 6B). Moreover, significant differences (P < 0.01) were found between the macropterous and the brachypterous brown plant hoppers in the numbers of eggs laid (Fig. 6B).

**Figure 7:**
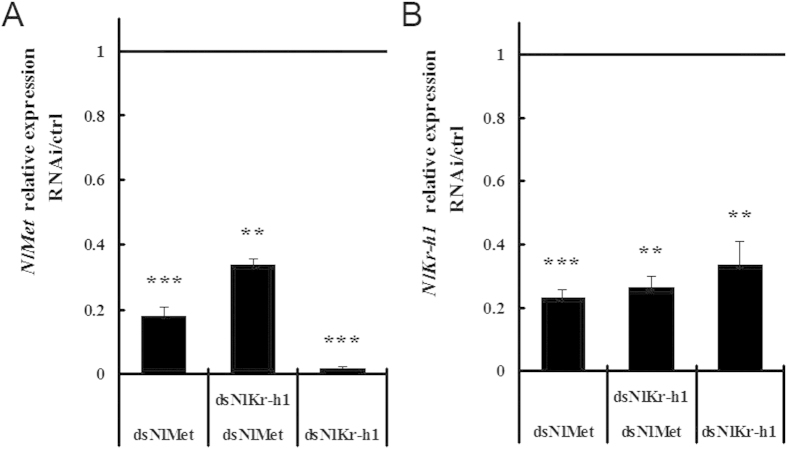
Relative expression levels of *NlMet* and *NlKr-h1* three days after gene silencing of *NlMet* or *NlKr-h1* or both in 5^th^ instar larvae of *N. lugens*. (**A**) *NlMet*; (**B**) *NlKr-h1*. The level of significance is based on t-tests, *P < 0.01, and error bars represent the standard error of the difference (SED).

Based on the qRT-PCR results, the expression of *NlMet* declined significantly in the dsNlMet-injected brown planthoppers (17.8%; Fig. 7A) and in the dsNlKr-h1 injected brown plant hoppers (1.53%, Fig. 7B), whereas the expression of *NlKr-h1* was reduced to 33.4% and 23.1% compared with the dsGFP-injected controls, respectively (Fig. 7). The expression levels of *NlMet* and *NlKr-h1* were reduced by 33.5% and 26.6%, respectively, upon the dual knockdown of *NlMet* and *NlKr-h1* in brown plant hoppers (Fig. 7).

## Discussion

### JH signalling and ovarian development

*NlMet* was expressed in all developmental stages, which was consistent with previous studies in which *Met* played a role during development and had a physiological function in *N. lugens*. The development of the ovarioles of *N. lugens* is affected by a variety of factors, including temperature and food availability^33^,^34^. Additionally, JH affects ovariole development in *N. lugens*^32^. The retarded development of the ovaries of female brown plant hoppers is typically caused by a deficiency of JH, and when JH is added in the required amount, the speed of ovary development recovers, even under conditions consisting of a lack of food and unfavourable temperatures^29^. JH plays an important role in oogenesis^35^,^36^, and JH is required for female reproductive maturation and vitellogenesis^35^,^36^,^37^. In a comparison with a study by Smykal *et al.*^38^, *Met* or *Tai* was required for the ovarian development of the linden bug, *Pyrrhocoris apterus*, whereas *Kr-h1* was not. According to Song *et al.*, however, *Kr-h1* was required for oocyte maturation and ovarian development in the migratory locust *Locusta migratoria*^22^.

In this study, in *N. lugens* females with disrupted JH signalling, the development of the ovarioles was affected, and abnormal phenotypes were observed (Fig. 3); moreover, we found that the progression through the stages of ovarian development declined significantly (Fig. 4), which indicated that JH signalling is required for the development of the ovary. Typically, the more highly evolved insects have longer preoviposition periods. In our experiment that measured the preoviposition period, the disruption of both *NlKr-h1* or *NlKr-h1* and *NlMet* significantly prolonged the preoviposition period, whereas the silencing of *NlMet* had no significant effect. This result indicated that *NlKr-h1* was required for the maturation of eggs in *N. lugens* but that *NlMet* was not required. In the *NlMet* knockdown or the *NlMet* and *NlKr-h1* dual knockdown brown plant hoppers, the ovaries developed unevenly (Fig. 3F,G). This result was similar to that in *D. menalogaster* in which a retinoic-like JH might be involved in genitalia rotation^39^, indicating that a similar signalling pathway might control the looping of left-right asymmetric organ formation.

### The interaction of *NlMet* and *NlKr-h1*

As previously mentioned, Met, along with its interaction partner Taiman, blocked ovarian development in the linden bug *Pyrrhocoris apterus*, although *Kr-h1* did not^21^. According to the current model of the JH signalling pathway, the transcription factor *Kr-h1* functions downstream of the JH receptor *Met*. The development of the ovarioles of dual knockdown *NlMet* and *NlKr-h1 N. lugens* brachypterous females was significantly slower than that in the adults treated separately with ds*NlMet* or ds*NlKr-h1*. This result suggests a possible interaction between *NlMet* and *NlKr-h1*. This interaction was more evident in the macropterous brown plant hoppers, i.e., silencing of neither *NlMet* nor *NlKr-h1* delayed ovariole development, whereas when both genes were silenced, ovariole development declined significantly (P < 0.01; Fig. 5B). Thus, when compared with the ovariole number and egg maturation, the development of the ovariole was more sensitive to the JH signalling.

### The migration of *N. lugens* and ovary development

Insect migration is an adaptive behaviour in response to environmental change and physiological factors. JH plays an important role in the migration and the reproduction of insects. A low level of JH typically stimulates insects to migrate, whereas a high level of JH stimulates the development of the ovary and the maturation of eggs. The macropterous form is typically responsible for long-distance migration and colonization of rice fields, whereas the brachypterous forms lay many more eggs than the macropterous forms. The role of JH in insect migration is complicated because in a dimorphic insect such as the brown plant hopper, the wing form is not only regulated by the levels of JH but also by the window of time for JH action, i.e., the 3^rd^ and the 4^th^ instar nymph stages are critical for the formation of wings. In the laboratory, the treatment of 3^rd^ and 4^th^ instar nymphs with JH significantly increased the proportion of brachypterous plant hoppers, whereas treatment with procene I increased the proportion of macropterous brown plant hoppers^32^. Notably, during migration, the ovary of *N. lugens* is typically underdeveloped, i.e., typically, before stage II^29^, and this observation indicates that JH affects both the wing form of the brown plant hopper and the reproductive state of the females. The earlier stage of ovarian development is an adaption of ovary development to migration because of the clear benefits obtained from an underdeveloped ovary for long-distance migration. The development of the ovaries of *N. lugens* continues after migration and colonization. The slowing of ovariole development after the silencing of JH signalling by RNAi targeting the JH receptor *NlMet* or its downstream transcription factor *NlKr-h1* is similar to that observed after treatment with antijuvenile hormone^29^,^32^. Therefore, in *N. lugens*, the role of JH in the development of wings and ovaries in females is clear. These results also explain, in part, the molecular mechanism of the role of JH in the migration of the brown plant hopper. Based on the results, JH is most likely an important factor in the regulation of ovarian development in *N. lugens*. In the future, the regulation of brown plant hopper populations through the molecular regulation of female reproduction and wing formation by modification of the JH signalling pathway will be possible.

## Materials and Methods

### Brown plant hopper

The brown plant hopper *Nilaparvata lugens* (Stål) was kindly provided by Professor Zengrong Zhu of Zhejiang University and was maintained in the laboratory. Seedlings of *Oryza sativa* L. cv. IIyou-203 were used to feed the brown plant hoppers at 25–28°C with a light:dark photoperiod of 14:10 h and humidity of 70–80%. To obtain sufficient numbers of brown plant hoppers for quantitative real-time PCR (qRT-PCR) and the RNA interference (RNAi) experiment, the insects were cultured asynchronously to ensure that all developmental stages were available simultaneously.

### RNA preparation, gene cloning and phylogenetic analysis

Total RNA was extracted from an equal mix of brown plant hopper nymphs and adults using the Trizol-based RNAiso Plus total RNA extraction kit (Takara, Dalian, China). First strand cDNA was synthesized using a Transcriptor First strand cDNA synthesis kit (Roche, Shanghai) and used as a template to amplify the gene of interest. The brown plant hopper homologue of *Met1* (*NlMet*) was searched in the transcriptome database. The full-length *NlMet* used for the synthesis of dsRNA was amplified by PCR using Ex-Taq polymerase (Takara, Dalian). The primers used were as follow: *NlMet*F: 5′CAACCAGCAGATGAACCTGA3′, and *NlMet*R: 5′GCAAAGCCTCGTACTCTTGG3′.

The PCR fragments were then purified with a gel purification kit (Omega, USA) and cloned into the PMD18-T vector (Takara, Dalian, China) for sequencing at Shanghai Sunny Biotechnology Co., Ltd. The sequences were aligned with ClustalW using Met homologues, and phylogenetic trees were constructed using MEGA6. For the sequence alignment and the phylogenetic tree, the search for homologues was performed using the Blast program at the NCBI (http://blast.ncbi.nlm.nih.gov).

### RNA extraction and qPCR

Quantitative Reverse Transcriptase PCR(qRT-PCR) was conducted using the Roche SYBR® Green PCR Master Mix and SYBR® Green RT-PCR Reagents Kit (Roche Applied Science, Shanghai, China). The reverse transcription was conducted as described by the supplier. The primers used were listed in Table 1.

A 25 μl reaction was used (Roche Applied Science, Shanghai), and 2 μl of diluted cDNA (20× dilution of the first strand cDNA synthesis reaction) was used for each reaction. A No Template Control (NTC) was used to monitor contamination and primer dimers. The 2^−ΔΔCt^ method^40^ was used to compare the relative levels of expression of the gene of interest at different developmental stages and in different tissues or before and after gene silencing. The reference genes used for qRT-PCR were previously reported^41^. Three biological replicates were used for the qRT-PCR experiment.

### RNAi experiment

For the RNAi experiment, the double-stranded RNA (dsRNA) was synthesized using the synthesis kit RiboMAX™ Large-Scale RNA Production System-T7 (Promega, Beijing). The templates for the synthesis of the dsRNA were amplified by PCR using the PMD18-T plasmid (Takara, Dalian, China) with a DNA fragment of the gene of interest inserted, and then the PCR products were purified with a DNA gel purification kit (Omega bio-tek, USA). The dsRNAs were synthesized as described in the Promega technical bulletin TB166. The primers used for the dsRNA synthesis of *NlMet*, *NlKr-h1* and the control gene encoding Green Fluorescent Protein (GFP) are listed in Table 2.

The 5^th^ instar nymphs were anaesthetized with CO_2_ before the intra-thoracic dsRNA injection. The injection was conducted using a Nikon microscope and a Narishige injection system (MN-151; Narishige). In total, 0.1 μg of dsRNA was injected into each insect. The nymphs were allowed to recover for 1–2 h after the injection and then were reared on rice seedlings. The mortality, wing-morph and sex were recorded after injection, and then the females were used for ovary dissections or were prepared for total RNA extraction. Three independent biological replicates were used for the nymphs that were injected with dsNlMet, dsNlKr-h1, both dsN1Met and dsN1Kr-h1, or only dsGFP.

### Ovary dissection and slide preparation

The female brown plant hoppers were anaesthetized using CO_2_ and were dissected in 1x PBS. The dissection was performed with a pair of tweezers, one to hold the thorax and the other to hold the lower part of the abdomen to gently tear the abdomen apart and expose the ovaries. The dissected ovaries were mounted in 70% glycerol on glass slides for further observation of the phenotypes or were prepared for total RNA extraction. The number of ovarioles was also recorded from counts under the microscope.

### Imaging and statistical analyses

The images of adult *N. lugens* and the ovaries from the gene silencing experiment were taken under a Nikon stereomicroscope (SMZ745T) and a Nikon fluorescence microscope (Eclipse 80i, 4X) with NIS-Elements. The images were processed with Adobe Photoshop CS5.

The statistical analyses were conducted using the SPSS 20 statistical software package. For the spatial and temporal expression of *NlMet*, significant differences were determined by one-way ANOVA and then Duncan’s multiple range tests. A Chi-square test was used for the comparison of the grading criteria for ovariole development.

## Additional Information

**How to cite this article**: Lin, X. *et al.* *Methoprene-tolerant (Met)* and *Krüpple-homologue* 1 *(Kr-h1)* are required for ovariole development and egg maturation in the brown plant hopper. *Sci. Rep.* **5**, 18064; doi: 10.1038/srep18064 (2015).

## Supplementary Material

Supplementary Figure 1

## Figures and Tables

**Table 1:** Primers for qRT-PCR.

Name	Forward (5′–3′)	Reverse (5′–3′)
*NlRPS11*	CCGATCGTGTGGCGTTGAAGGG	ATGGCCGACATTCTTCCAGGTCC
*NlMet*	GGTGGTAAACGGATTGGAAA	CATCGTCAGCCAACTCGATA
*NlKr-h1*	TGATGAGGCACACGATGACT	ATGGAAGGCCACATCAAGAG

**Table 2:** Primers for dsRNA Synthesis.

Name	Nucleotide sequence (5′–3′)
dsNlMetT7F	TAATACGACTCACTATAGGGAGACCACCAACCAGCAGATGAACCTGA
dsNlMetT7R	TAATACGACTCACTATAGGGAGACCACGCAAAGCCTCGTACTCTTGG
dsNlKrhT7F	TAATACGACTCACTATAGGGAGACCACGTGGGGTTCAGTCCTGAGGA
dsNlKrhT7R	TAATACGACTCACTATAGGGAGACCACCAGTCGAACACACACCGGAG
dsGFPT7F	GGATCCTAATACGACTCACTATAGGAAGGGCGAGGAGCTGTTCACCG
dsGFPT7R	GGATCCTAATACGACTCACTATAGGCAGCAGGACCATGTGATCGCGC

## References

[b1] Hu G. *et al.* Outbreaks of the brown planthopper Nilaparvata lugens (Stal) in the Yangtze River Delta: immigration or local reproduction? PLoS One 9, e88973, 10.1371/journal.pone.0088973 (2014). 24558459 PMC3928339

[b2] Hu G. *et al.* Rice planting systems, global warming and outbreaks of Nilaparvata lugens (Stal). Bull Entomol Res 101, 187–199, 10.1017/S0007485310000313 (2011). 20961467

[b3] Yang W., Wang X., Wang S., Yie Y. & Tien P. Infection and replication of a planthopper transmitted virus-rice stripe virus in rice protoplasts. J Virol Methods 59, 57–60 (1996). 8793830 10.1016/0166-0934(96)02013-7

[b4] Chomchan P., Miranda G. J. & Shirako Y. Detection of rice grassy stunt tenuivirus nonstructural proteins p2, p5 and p6 from infected rice plants and from viruliferous brown planthoppers. Arch Virol 147, 2291–2300, 10.1007/s00705-002-0893-4 (2002). 12491098

[b5] Jindra M., Palli S. R. & Riddiford L. M. The juvenile hormone signaling pathway in insect development. Annu Rev Entomol 58, 181–204, 10.1146/annurev-ento-120811-153700 (2013). 22994547

[b6] Charles J. P. *et al.* Ligand-binding properties of a juvenile hormone receptor, Methoprene-tolerant. Proc Natl Acad Sci USA 108, 21128–21133, 10.1073/pnas.1116123109 (2011). 22167806 PMC3248530

[b7] Li M., Mead E. A. & Zhu J. Heterodimer of two bHLH-PAS proteins mediates juvenile hormone-induced gene expression. Proc Natl Acad Sci USA 108, 638–643, 10.1073/pnas.1013914108 (2011). 21187375 PMC3021087

[b8] Zhang Z., Xu J., Sheng Z., Sui Y. & Palli S. R. Steroid receptor co-activator is required for juvenile hormone signal transduction through a bHLH-PAS transcription factor, methoprene tolerant. J Biol Chem 286, 8437–8447, 10.1074/jbc.M110.191684 (2011). 21190938 PMC3048728

[b9] Li M. *et al.* A steroid receptor coactivator acts as the DNA-binding partner of the methoprene-tolerant protein in regulating juvenile hormone response genes. Mol Cell Endocrinol 394, 47–58, 10.1016/j.mce.2014.06.021 (2014). 25004255 PMC4163509

[b10] Shin S. W., Zou Z., Saha T. T. & Raikhel A. S. bHLH-PAS heterodimer of methoprene-tolerant and Cycle mediates circadian expression of juvenile hormone-induced mosquito genes. Proc Natl Acad Sci USA 109, 16576–16581, 10.1073/pnas.1214209109 (2012). 23012454 PMC3478602

[b11] Jin M. N., Xue J., Yao Y. & Lin X. D. Molecular Characterization and Functional Analysis of Krüppel-homolog 1 (Kr-h1) in the Brown Planthopper, Nilaparvata lugens (Stål). Journal of Integrative Agriculture 13, 1972–1981 (2014).

[b12] Fichelson P., Brigui A. & Pichaud F. Orthodenticle and Kruppel homolog 1 regulate Drosophila photoreceptor maturation. Proc Natl Acad Sci USA 109, 7893–7898, 10.1073/pnas.1120276109 (2012). 22547825 PMC3356647

[b13] Shi L. *et al.* Roles of Drosophila Kruppel-homolog 1 in neuronal morphogenesis. Dev Neurobiol 67, 1614–1626, 10.1002/dneu.20537 (2007). 17562531

[b14] Hepat R. & Kim Y. JH modulates a cellular immunity of Tribolium castaneum in a Met-independent manner. J Insect Physiol 63, 40–47, 10.1016/j.jinsphys.2014.02.008 (2014). 24607640

[b15] Xu J., Sheng Z. & Palli S. R. Juvenile hormone and insulin regulate trehalose homeostasis in the red flour beetle, Tribolium castaneum. PLoS Genet 9, e1003535, 10.1371/journal.pgen.1003535 (2013). 23754959 PMC3675034

[b16] Bilen J., Atallah J., Azanchi R., Levine J. D. & Riddiford L. M. Regulation of onset of female mating and sex pheromone production by juvenile hormone in Drosophila melanogaster. Proc Natl Acad Sci USA 110, 18321–18326, 10.1073/pnas.1318119110 (2013). 24145432 PMC3831481

[b17] Wilson T. G., DeMoor S. & Lei J. Juvenile hormone involvement in Drosophila melanogaster male reproduction as suggested by the Methoprene-tolerant(27) mutant phenotype. Insect Biochem Mol Biol 33, 1167–1175, S0965174803001498 [pii] (2003). 14599489 10.1016/j.ibmb.2003.06.007

[b18] Riddiford L. M. How does juvenile hormone control insect metamorphosis and reproduction? Gen Comp Endocrinol 179, 477–484, 10.1016/j.ygcen.2012.06.001 (2012). 22728566

[b19] Parthasarathy R., Sheng Z., Sun Z. & Palli S. R. Ecdysteroid regulation of ovarian growth and oocyte maturation in the red flour beetle, Tribolium castaneum. Insect Biochem Mol Biol 40, 429–439, 10.1016/j.ibmb.2010.04.002 (2010). 20385235 PMC2916939

[b20] Zou Z. *et al.* Juvenile hormone and its receptor, methoprene-tolerant, control the dynamics of mosquito gene expression. Proc Natl Acad Sci USA 110, E2173–2181, 10.1073/pnas.1305293110 (2013). 23633570 PMC3683779

[b21] Smykal V. *et al.* Juvenile hormone signaling during reproduction and development of the linden bug, Pyrrhocoris apterus. Insect Biochem Mol Biol 45, 69–76, 10.1016/j.ibmb.2013.12.003 (2014). 24361539

[b22] Song J., Wu Z., Wang Z., Deng S. & Zhou S. Kruppel-homolog 1 mediates juvenile hormone action to promote vitellogenesis and oocyte maturation in the migratory locust. Insect Biochem Mol Biol 52, 94–101, 10.1016/j.ibmb.2014.07.001 (2014). 25017142

[b23] Konopova B., Smykal V. & Jindra M. Common and distinct roles of juvenile hormone signaling genes in metamorphosis of holometabolous and hemimetabolous insects. PLoS One 6, e28728, 10.1371/journal.pone.0028728 (2011). 22174880 PMC3234286

[b24] Kayukawa T. *et al.* Transcriptional regulation of juvenile hormone-mediated induction of Kruppel homolog 1, a repressor of insect metamorphosis. Proc Natl Acad Sci USA 109, 11729–11734, 10.1073/pnas.1204951109 (2012). 22753472 PMC3406821

[b25] Kayukawa T. *et al.* Hormonal regulation and developmental role of Kruppel homolog 1, a repressor of metamorphosis, in the silkworm Bombyx mori. Dev Biol 388, 48–56, 10.1016/j.ydbio.2014.01.022 (2014). 24508345

[b26] Minakuchi C., Zhou X. & Riddiford L. M. Kruppel homolog 1 (Kr-h1) mediates juvenile hormone action during metamorphosis of Drosophila melanogaster. Mech Dev 125, 91–105, 10.1016/j.mod.2007.10.002 (2008). 18036785 PMC2276646

[b27] Minakuchi C., Namiki T. & Shinoda T. Kruppel homolog 1, an early juvenile hormone-response gene downstream of Methoprene-tolerant, mediates its anti-metamorphic action in the red flour beetle Tribolium castaneum. Dev Biol 325, 341–350, 10.1016/j.ydbio.2008.10.016 (2009). 19013451

[b28] Cui Y., Sui Y., Xu J., Zhu F. & Palli S. R. Juvenile hormone regulates Aedes aegypti Kruppel homolog 1 through a conserved E box motif. Insect Biochem Mol Biol 52, 23–32, 10.1016/j.ibmb.2014.05.009 (2014). 24931431 PMC4143451

[b29] Chen J. C., Cheng S. N., Yan L. M. & Yin H. The ovarial development of the brown planthopper(Nilaparvat lugens stal) and it’s relation to migration. Acta Entomologica Sinica 22, 280–288 (1979).

[b30] Lu F. *et al.* The processes of morphological change and grading criteria for ovariandevelopment in the brown planthopper. Chinese Journal of Applied Entomology 48, 1394–1400 (2011).

[b31] Chen Y. *et al.* The effects of rice growth stages on the ovarian development and take-off of Nilaparvata lugens and Sogatella furcifera. Acta Ecologica Sinica 32, 1546–1552 (2012).

[b32] Iwanaga K. & S. T. Effects of juvenile hormone and rearing density on wing dimorphism and oöcyte development in the brown planthopper, Nilaparvata lugens. Journal of Insect Physiology 32, 585–590 (1986).

[b33] Li R. D. Population growth of the brown planthopper, Nilaparvata lugens stål, as influenced by temperature. Acta Phytophylacica Sinica 11, 101–108 (1984).

[b34] Cheng J. A. & Zhu Z. R. Analysis on the key factors causing the outbreak of brown planthopper in Yangtze area, China in 2005. Plant Protection 32, 104 (2006).

[b35] Dubrovsky E. B., Dubrovskaya V. A. & Berger E. M. Juvenile hormone signaling during oogenesis in Drosophila melanogaster. Insect Biochem Mol Biol 32, 1555–1565 (2002). 12530223 10.1016/s0965-1748(02)00076-0

[b36] Pound J. M. & Oliver J. H. Jr. Juvenile hormone: evidence of its role in the reproduction of ticks. Science 206, 355–357, 10.1126/science.206.4416.355 (1979). 17733685

[b37] Sun G., Zhu J., Chen L. & Raikhel A. S. Synergistic action of E74B and ecdysteroid receptor in activating a 20-hydroxyecdysone effector gene. Proc Natl Acad Sci USA 102, 15506–15511, 10.1073/pnas.0503501102 (2005). 16230625 PMC1266084

[b38] Smykal V. *et al.* Importance of juvenile hormone signaling arises with competence of insect larvae to metamorphose. Dev Biol 390, 221–230, 10.1016/j.ydbio.2014.03.006 (2014). 24662045

[b39] Adam G., Perrimon N. & Noselli S. The retinoic-like juvenile hormone controls the looping of left-right asymmetric organs in Drosophila. Development 130, 2397–2406 (2003). 12702654 10.1242/dev.00460

[b40] Livak K. J. & Schmittgen T. D. Analysis of relative gene expression data using real-time quantitative PCR and the 2(-Delta Delta C(T)) Method. Methods 25, 402–408, 10.1006/meth.2001.1262 (2001). 11846609

[b41] Yuan M. *et al.* Selection and evaluation of potential reference genes for gene expression analysis in the brown planthopper, Nilaparvata lugens (Hemiptera: Delphacidae) using reverse-transcription quantitative PCR. PLoS One 9, e86503, 10.1371/journal.pone.0086503 (2014). 24466124 PMC3900570

