# Supplementary material for: Methoprene-tolerant (Met) and Krüpple-homologue 1 (Kr-h1) are required for ovariole development and egg maturation in the brown plant hopper
Source: Sci Rep. 2015 Dec 14;5:18064. doi: 10.1038/srep18064 (PMC4677288; doi:10.1038/srep18064)
Supplement: Supplementary Figure 1 [file srep18064-s1.doc]

**Title:**

*Methoprene-tolerant* (*Met*) and *Krüpple-homologue* 1 (*Kr-h1*) are required for ovariole development and egg maturation in the brown plant hopper

Xinda Lin*, Yun Yao, Bo Wang

**Author affiliation:**

College of Life Sciences, China Jiliang University, Hangzhou, China, 310018

***Corresponding author：**

Xinda Lin, College of Life Sciences, China Jiliang University, Hangzhou, China, 310018

Telephone: +86-139-5802-8822

E-mail: linxinda@cjlu.edu.cn

**Supplementary Material**


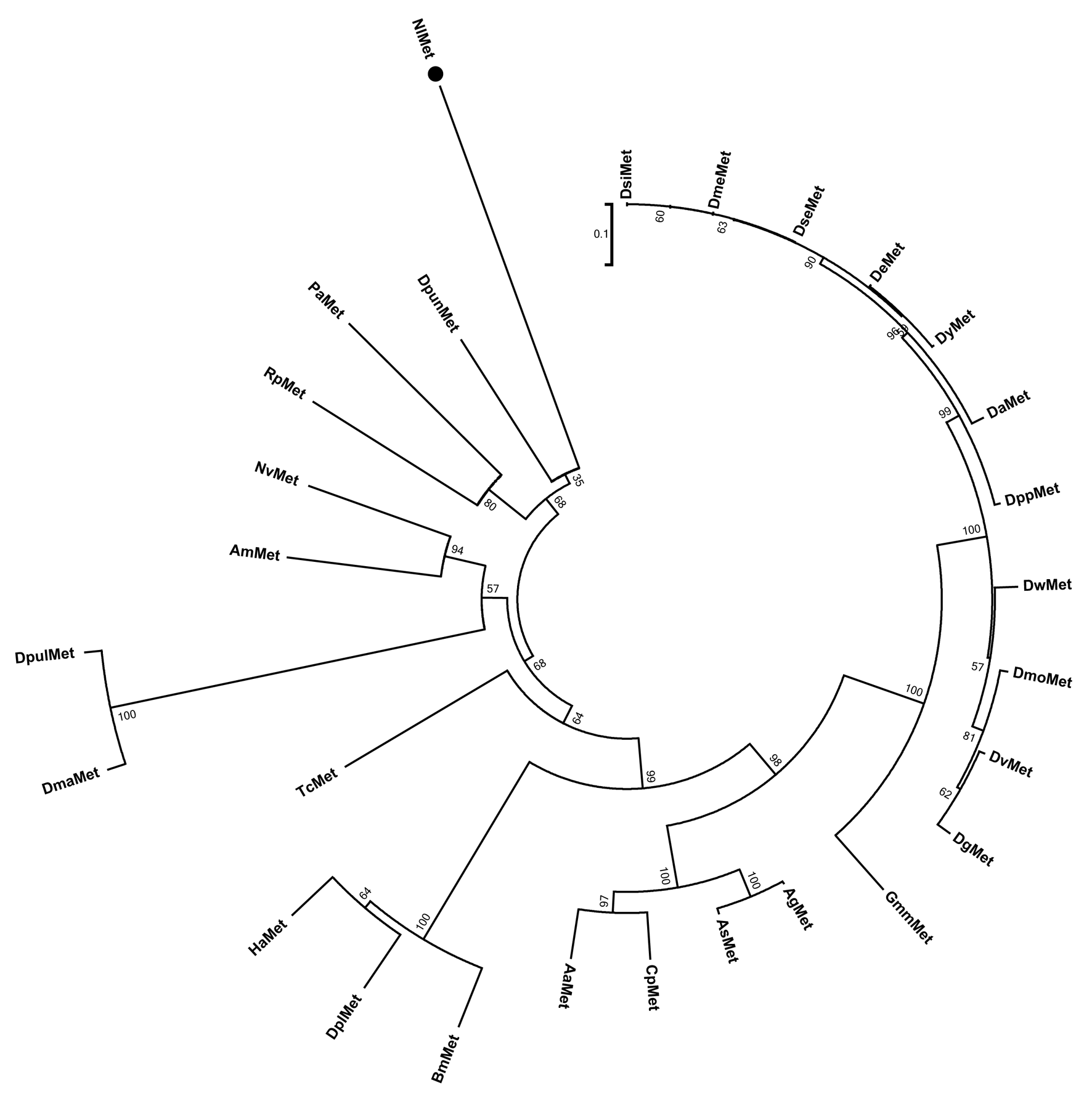


**Supplementary Figure 1: Phylogenetic analyses of the *Met* homologues.**

Aa, *Aedes aegypti* (AAW82472.1); Ag, *Anopheles gambiae* (ABC18327.1); Am, *Apis mellifera* (XP_395005.4); As, *Anopheles sinensis* (KFB43800.1); Bm, *Bombyx mori* (ACJ04052.1); Cp, *Culex pipiens* (AAW81958.1); Da, *Drosophila erecta* (Drosophila erecta); De, *Drosophila erecta* (XP_001977095.1); Dg, *Drosophila grimshawi* (XP_001992460.1); Dme, *Drosophila melanogaster* (NP_511126.2); Dma, *Daphnia magna* (BAM83855.1); Dmo, *Drosophila mojavensis* (XP_002010196.1); Dpl, *Danaus plexippus* (EHJ75902.1); Dpul, *Daphnia pulex* (BAM83853.1); DpunMet, *Diploptera punctata* (AIM47235.1); Dpp, *Drosophila pseudoobscura pseudoobscura* (XP_001355648.2); Dse, *Drosophila sechellia* (XP_002044104.1); Dsi, *Drosophila simulans* (XP_002106697.1); Dv, *Drosophila virilis* (XP_002055682.1); Dw, *Drosophila willistoni* (XP_002071666.1); Dy, *Drosophila yakuba* (XP_002101394.1); Gmm, *Glossina morsitans morsitans* (AFQ01087.1); Ha, *Helicoverpa armigera* (AHX26585.1); Nl, *Nilaparvata lugens* (AIE12451.1); Nv, *Nasonia vitripennis* (XP_001606775.2); Rp, *Rhodnius prolixus* (AEW22977.1); Pa, *Pyrrhocoris apterus* (AEW22976.1); Tc, *Tribolium castaneum* (NP_001092812.1).
